# Immune correlates in a Phase II clinical trial with ipilimumab in combination with finite androgen deprivation therapy in patients with metastatic non-castrate prostate cancer

**DOI:** 10.1186/2051-1426-3-S2-P106

**Published:** 2015-11-04

**Authors:** Sumit K Subudhi, Ana Aparicio, Jianjun Gao, Amado Zurita, Araujo John, Christopher Logothetis, Brinda Rao, Luis Vence, James Allison, Ryan O Emerson, Erik Yusko, Marissa Vignali, Harlan Robins, Jing Jing Sun, Padmanee Sharma

**Affiliations:** 1 The University of Texas MD Anderson Cancer Center, Houston, TX, USA; 2 Adaptive Biotechnologies Corporation, Seattle, WA, USA

## Background

Ipilimumab is an immunotherapy that blocks the function of an immune checkpoint, cytotoxic T-lymphocyte antigen 4 (CTLA-4), and promotes durable anti-tumor responses in a subset of patients. We hypothesized that ipilimumab could be added to androgen deprivation therapy (ADT) as treatment for patients with metastatic prostate cancer in an attempt to limit the amount of ADT (8 months) but provide durable anti-tumor responses. We aimed to correlate immunologic biomarkers with clinical responses, including immune-related adverse events (irAEs).

## Methods

We conducted a single-arm, single-center, observational study from July, 2011 to June, 2013 to determine if finite ADT plus ipilimumab is safe, estimate efficacy and link clinical outcomes to immunologic changes in men with metastatic non-castrate prostate cancer. ADT was administered for only 8 months with concurrent ipilimumab (10 mg/kg) for up to 4 doses at 4-weeks intervals.

## Results

We screened 30 pts of which 24 were evaluable for efficacy and 27 for safety. The median time to PSA progression was 9.9 (IQR 5.8-13.1) months from treatment initiation, and two of the 24 (8%) patients have PSA responses that are ongoing for 40.7 and 17.9 months, respectively. The trial was closed as pre-specified due to excess toxicity in which 12 of 27 (44%) patients developed grade 3 immune-related adverse events (irAEs), which have been observed with Ipilimumab monotherapy. The most common grade 3 events were transaminitis, colitis/diarrhea, and hypophysitis. Immunological biomarkers were identified that potentially predict for clinical benefit and grade 3 irAEs.

## Conclusions

This pilot study suggests the combination of ipilimumab plus finite ADT can induce durable responses in a subset of patients with metastatic prostate cancer. irAEs are likely to occur and require careful management. Comprehensive immune monitoring of peripheral blood CD4 and CD8 T cells by flow cytometry and sequencing the CDR3 region of the T cell receptor beta chain revealed potential biomarkers predictive of clinical outcomes. Future studies with lower doses of ipilimumab may need to be tested in combination with ADT.

